# Pathogenic mechanisms of preeclampsia with severe features implied by the plasma exosomal mirna profile

**DOI:** 10.1080/21655979.2021.1993717

**Published:** 2021-12-09

**Authors:** Zhirui Chen, Wen Zhang, Mengying Wu, Haixia Huang, Li Zou, Qingqing Luo

**Affiliations:** a Department of Obstetrics, The Affiliated Hospital of Southwest Medical University, Luzhou, China; b Department of Obstetrics and Gynecology, Union Hospital, Tongji Medical College, Huazhong University of Science and Technology, Wuhan, Hubei, China; c Department of Obstetrics, Wuhan Children’s Hospital (Wuhan Maternal and Child Healthcare Hospital), Tongji Medical College, Huazhong University of Science and Technology, Wuhan, Hubei, China

**Keywords:** exosomes, miRNAs profile, pre-eclampsia, gene ontology, KEGG, endothelial cell dysfunction

## Abstract

Preeclampsia is a complication of pregnancy characterized by high blood pressure and organ damage after 20 gestational weeks. It is associated with high maternal and fetal morbidity and mortality. However, at present, there is no effective prevention or treatment for this condition. Previous studies have revealed that plasma exosomal mirnas from pregnant women with preeclampsia could serve as biomarkers of pathogenic factors. However, the roles of plasma exosomal mirnas in preeclampsia with severe features (sPE), which is associated with poorer pregnancy outcomes, remain unknown. Thus, the aims of this study were to characterize plasma exosomal miRNAs in sPE and explore the related pathogenic mechanisms using bioinformatic analysis. Plasma exosomes were isolated using a mirVana RNA isolation kit. the exosomal miRNAs were detected using high-throughput sequencing and the mirnas related to Kyoto Encyclopedia of Genes and Genomes (KEGG) pathways and gene ontology (GO) terms were analyzed using the clusterprofiler package of R. Fifteen miRNAs exhibited increased expression and fourteen miRNAs exhibited reduced expression in plasma exosomes from women with sPE as compared to normal pregnant women. Further, gene set enrichment analysis revealed that the differentially expressed plasma exosomal miRNAs were related to the stress response and cell junction regulation, among others. In summary, this study is the first to identify the differentially expressed plasma exosomal miRNAs in sPE. These findings highlight promising pathogenesis mechanisms underlying preeclampsia.

## Introduction

Preeclampsia is a common complication of pregnancy that affects 2-8% of pregnant women worldwide [1]. Preeclampsia is associated with high maternal and fetal morbidity and mortality as well as an increased incidence of cardiovascular diseases in later life for mothers and an increased incidence of childhood allergies among their children [2]. Preeclampsia with severe features (sPE) is associated with even poorer outcomes and is diagnosed when the pregnant woman’s blood pressure is over 160/110 mmHg or she experiences other severe features such as thrombocytopenia, impaired liver function or renal insufficiency [1]. Although the pathogenesis of preeclampsia remains under debate, the two-stage theory is most widely accepted. According to the two-stage theory, abnormal placentation and subsequent placental hypoperfusion resulting from inadequate trophoblast invasion lead to local oxidative stress and excessive inflammation at the maternal-fetal interface. Then, the injured placenta secretes soluble pathogenic factors, such as soluble fms-like tyrosine kinase-1 (sFlt-l) and soluble endoglin (sENG), into the mother’s circulation, causing impairment in the maternal organs and leading to the clinical manifestations of preeclampsia [3]. Thus, effective recognition of placenta impairment in the early stage of preeclampsia will guide the timely application of interventions to restrict the process of sPE. However, at present, there is no effective method for the early identification of patients at high risk of preeclampsia in clinical practice. Therefore, exploration of effective early identification approaches is of great significance for disease prevention and treatment.

Exosomes are cell-derived nanoscale extracellular vesicles (40-150 nm in diameter) that widely exist in almost all bodily fluids [4]. Containing multiple biomolecules, such as proteins, glycans, lipids, nucleic acids and metabolites, exosomes have attracted considerable attention as disease biomarkers and intercellular communicators [4,5]. Due to their stability and safety, exosomes have great potential as therapeutic carriers in vaccines, chemical drugs, fungal-derived materials and so on [6,7]. During pregnancy, the concentration of total exosomes in the serum of pregnant women dramatically increases, suggesting that serum exosomes may play special roles during pregnancy [8]. Many studies have indicated that plasma exosomes in pregnant women can deliver pathogenic molecules such as sFlt-1 and circular RNAs and carry disease biomarkers such as micro RNAs [9,10]. MiRNAs, small non-coding RNA fragments (about 20 bp in length), have been widely studied as regulators of target gene and disease biomarkers that can be collected easily and detected rapidly [11,12]. As important cargo of exosomes, exosomal miRNAs exist in the circulation with the protection of the exosomal membrane [13]. Although several studies have detected different plasma exosomal miRNA profiles between women with preeclampsia and normal pregnant women, few studies have focused on exosomal miRNAs in sPE (Table 1) [14–17], which limits our understanding of this disease.

**Table 1.** Studies profiling the plasma exosomal miRNAs of patients with preeclampsia. PE: preeclampsia, EOPE: early onset preeclampsia, LOPE: late onset preeclampsia

Literatures	Group design	Detection method	Main findings
Salomon et al. (2017) [14]	PE vs. normal	Next-generation sequencing	Identified 12 differentially expressed serum exosomal miRNAs between normal group and PE group.
Pillay et al. (2019) [15]	EOPE/LOPE vs. normal	Next-generation sequencing	Identified differentially expressed serum exosomal miRNAs in early and late onset preeclampsia, such as miR-3605-3p and miR-122-5p.
Devor et al. (2020) [16]	PE vs. normal	TaqMan Low Density microRNA Arrays (TLDA)	Detected exosomal miRNAs profiles in each trimester of pregnancy, provided biomarkers of predictive value for preeclampsia.
Li et al. (2020) [17]	PE vs. normal	qPCR-based TaqMan Advanced miRNA assay	Found 7 miRNA species were differentially expressed in serum exosomes from women with preeclampsia and those from controls.

Therefore, the primary aim of this study was to detect differences in the plasma exosomal miRNA profiles between normal pregnant women and those with sPE. Subsequently, bioinformatics analysis of the differentially expressed exosomal miRNAs was performed to predict target genes and identify their potential functions in sPE, revealing the possible pathogenic mechanisms of this disease.

## Materials and methods

The control group (normotensive pregnant woman, n=3) and sPE group (patients with sPE, n=3) were recruited from the Department of Obstetrics and Gynecology, Union Hospital, Tongji Medical College, Huazhong University of Science and Technology. The clinical characteristics of the patients are presented in Table 2. Pregnant women with severe hypertension (blood pressure over 160/110 mmHg) or other features (thrombocytopenia, renal insufficiency, impaired liver function, new-onset headache, visual disturbances and pulmonary edema) were diagnosed with sPE [1]. All pregnancies were singleton without any medical, surgical or other obstetric complications. Pregnant women with a history of obstetric complications were also excluded from the control group. The study was approved by the ethics committee of Tongji Medical College, Huazhong University of Science and Technology (HUST) (No. 2018S419) and was performed in accordance with the Declaration of Helsinki. Maternal venous blood was collected into EDTA-anticoagulant tubes and stored at 4°C until centrifugation. Plasma was separated by centrifugation at 3,000g for 15 min at room temperature and then stored at -80°C until exosome isolation.

**Table 2.** Clinical characteristics of enrolled patients. *: *p*-value<0.05. ALT: alanine aminotransferase; AST: aspartate aminotransferase

	control(n=3)	sPE(n=3)
Age (y)	29.67±1.70	28.67±2.05
Body mass index(kg/m^2^)	25.30±2.36	24.78±2.19
Gestational week (weeks)	39.00±0.82	33.33±2.36
Systolic pressure(mmHg)	125.00±4.08	163.33±4.71*
Diastolic pressure(mmHg)	71.67±6.24	112.67±12.28*
ALT (U/L)	24.33±13.70	269.00±347.20
AST (U/L)	23.33±5.56	227.67±288.74
Platelet (10^9^/L)	203.67±43.28	133.33±2.62
Proteinuria positive ratio (%)	0	100
Apgar score at1min	9±0	7±0.82
Neonatal birth weight (g)	3150.00±294.40	1766.67±572.03*

**Table 3.** Differentially expressed plasma exosomal miRNAs

miRNA	log2FoldChange	*P*-value	Sequence (5ʹ to 3ʹ)
hsa-miR-125a-3p	-2.909723858	0.023170976	ACAGGTGAGGTTCTTGGGAGCC
hsa-miR-125b-5p	2.360338783	0.025220126	TCCCTGAGACCCTAACTTGTGA
hsa-miR-148a-5p	-3.297554546	0.004103378	AAAGTTCTGAGACACTCCGACT
hsa-miR-155-5p	3.443051698	0.001495107	TTAATGCTAATCGTGATAGGGGTT
hsa-miR-17-5p	2.987587066	0.012174407	CAAAGTGCTTACAGTGCAGGTAG
hsa-miR-193b-5p	-2.977244619	0.028855453	CGGGGTTTTGAGGGCGAGATGA
hsa-miR-195-3p	-3.45566586	0.018709016	CCAATATTGGCTGTGCTGCTCC
hsa-miR-199a-5p	2.186004643	0.017650947	CCCAGTGTTCAGACTACCTGTTC
hsa-miR-200c-3p	5.480264965	8.13E-05	TAATACTGCCGGGTAATGATGGA
hsa-miR-203a-3p	3.841681468	0.005200292	GTGAAATGTTTAGGACCACTAG
hsa-miR-205-5p	3.838203786	0.009743346	TCCTTCATTCCACCGGAGTCTG
hsa-miR-206	-3.311518297	0.008274703	TGGAATGTAAGGAAGTGTGTGG
hsa-miR-2110	-5.070758573	0.000654162	TTGGGGAAACGGCCGCTGAGTG
hsa-miR-215-5p	4.459820445	0.001085737	ATGACCTATGAATTGACAGAC
hsa-miR-328-3p	3.34533119	0.033574838	CTGGCCCTCTCTGCCCTTCCGT
hsa-miR-331-5p	-5.205950072	0.000989231	CTAGGTATGGTCCCAGGGATCC
hsa-miR-335-5p	-2.109330178	0.0415164	TCAAGAGCAATAACGAAAAATGT
hsa-miR-340-5p	2.366380449	0.048545552	TTATAAAGCAATGAGACTGATT
hsa-miR-370-3p	3.254431927	0.00836901	GCCTGCTGGGGTGGAACCTGGT
hsa-miR-483-5p	3.447628353	0.0109226	AAGACGGGAGGAAAGAAGGGAG
hsa-miR-500a-3p	-2.480551744	0.034688244	ATGCACCTGGGCAAGGATTCTG
hsa-miR-509-3p	2.596975968	0.031922797	TGATTGGTACGTCTGTGGGTAG
hsa-miR-518b	3.306397487	0.012835174	CAAAGCGCTCCCCTTTAGAGGT
hsa-miR-543	-2.165628912	0.032301788	AAACATTCGCGGTGCACTTCTT
hsa-miR-548o-3p	3.342808425	0.016714456	CCAAAACTGCAGTTACTTTTGC
hsa-miR-654-5p	-2.300317719	0.026300428	TGGTGGGCCGCAGAACATGTGC
hsa-miR-660-5p	-5.685021088	0.000117126	TACCCATTGCATATCGGAGTTG
hsa-miR-744-5p	-2.70869948	0.04381084	TGCGGGGCTAGGGCTAACAGCA
hsa-miR-9985	-3.609719989	0.002649267	TTCACAGTGGCTAAGCTAT

### Exosome isolation and identification

Plasma exosomes were isolated by ultracentrifugation, as described previously [18]. Every 1 ml of plasma was diluted in 25 ml phosphate-buffered saline (PBS). After centrifugation twice at low speed (300 g for 10 min and 2000 g for 10 min), the supernatant was collected and centrifuged again at 10,000 g for 30 min, and then was filtered through a 0.22 µm pore size membrane filter. The pre-processed samples were ultracentrifuged at 120,000 g for 75 min and the pelleted fraction was washed with PBS before being ultracentrifuged again. Finally, the supernatant was removed and the pelleted fraction containing exosomes was re-suspended.

The particle size and concentration of exosomes were analysed with nanoparticle tracking analysis (NTA) using ZetaView PMX 110 (Particle Metrix, Meerbusch, Germany) and the corresponding software ZetaView 8.04.02, as described previously [19]. The morphology of the isolated exosomes was characterised by transmission electron microscopy (TEM) under a Hitachi TEM system (Hitachi High-Technologies Corporation, Japan), as previously described [20]. The specific proteins of the isolated exosomes were analysed by western blot. Briefly, 30 μg of protein from each sample was separated by 12% sodium dodecyl sulphate-polyacrylamide gel electrophoresis before being transferred onto nitrocellulose membranes. The membranes were incubated with three primary antibodies, anti-CD63 (Proteintech, #25682-1-AP, 1:500), anti-CD9 (ab92726, Abcam, United Kingdom, 1:1000) and placental alkaline phosphatase (PLAP) antibody (ab133602, Abcam, United Kingdom, 1:1000) overnight at 4 C. Then, they were incubated with the secondary antibody (Biosharp, #70090100, 1:10000) for 2 h at room temperature. The proteins were visualised by the enhanced chemiluminescence (ECL) procedure.

### Exosomal RNA extraction and sequencing

Total RNA was isolated from exosomes using a mirVana RNA isolation kit (Ambion-1561, Austin, TX, USA), according to the manufacturer’s recommendations with modifications [21]. Illumina TruSeq Small RNA Library Preparation Kits (Cat. No. RS-200-0012, Illumina, USA) were used to prepare the miRNA libraries and miRNA sequencing was performed on an Illumina HiSeq 2000 (Illumina, San Diego, CA).

### Bioinformatics analysis

Differential expression of the sequencing data was determined with the DESeq2 R package (version 1.30.0) based on the following cut-off criteria: *p*-value<0.05 and abstract Log2 (fold change). The sequencing data have been deposited in the Gene Expression Omnibus database under GSE175807. The targets of exosomal miRNAs were identified by two online databases: Targetscan (http://www.targetscan.org/) and miRDB (http://mirdb.org/). Gene ontology (GO) analysis and Kyoto encyclopedia of genes and genomes (KEGG) pathway analysis were performed using the clusterProfiler R package (version 3.18.0) with an adjusted *p*-value < 10^−4^.

### Statistical analysis

Clinical data were compared using unpaired Student’s *t*-tests with Welch’s correction. Analyses were performed in Prism 5 and quantitative data are expressed as the mean ± standard deviation. A *p*-value < 0.05 was considered statistically significant.

## Results

To explore the roles of plasma exosomal miRNAs in the development of sPE, plasma exosomes were collected from normal pregnant women and patients with sPE. Then, the plasma exosomal miRNA profiles were detected using high-throughput sequencing. The differentially expressed miRNAs were identified with differential expression analysis and their predicted target genes were identified by enrichment analysis.

### Characterization of plasma exosomes

**Figure 1.**
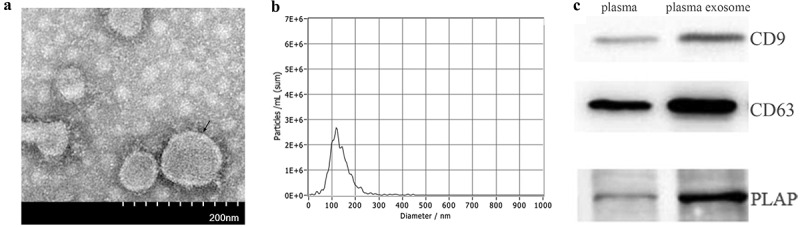
Identification of isolated plasma exosomes. (a) TEM of exosomes. black arrow: exosomes. (b) particle diameters distribution of exosomes. (c) western blot of exosomal markers

Isolated exosomes exhibited a cup-like morphology (Figure 1(a)). NTA measurement showed that the particle diameters primarily ranged from 30 to 200 nm (Figure 1(b)) with a peak particle size of 120 nm. Western blot showed positive expression of typical exosomal markers, CD9 and CD63, and placenta-derived particle, PLAP, in isolated exosomes (Figure 1(c)).

### Different expression of exosomal miRNAs

**Figure 2.**
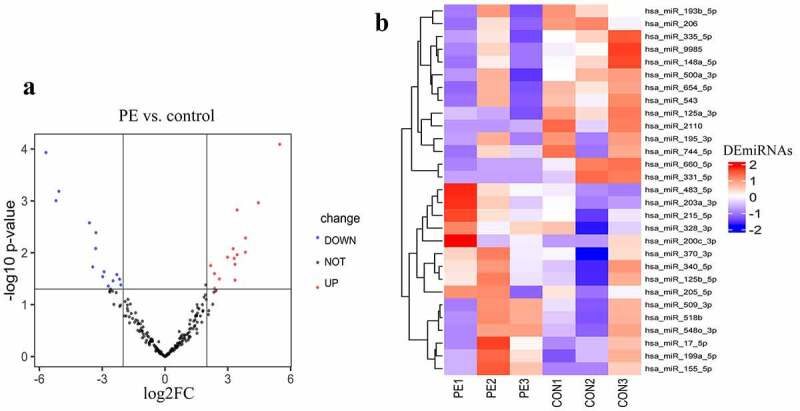
Differentially expression analysis. (a) volcano plot of differentially expressed miRNAs. (b) heat map of differentially expressed miRNAs

High-throughput sequencing detected 797 known miRNAs. In total, 29 differentially expressed exosomal miRNAs were identified between sPE and controls, among which, 15 miRNAs had increased expression (UEMir) and 14 miRNAs had decreased expression (DEMir) in sPE (Figure 2(a,b) and Table 3). Given that exosomal miRNAs with low abundance are unable to have a considerable regulatory effect, miRNAs with detected counts <10 were excluded in the following gene set enrichment analysis. As a result, 7 UEMirs (miR-125b-5p, miR-199a-5p, miR-200c-3p, miR-203a-3p, miR-215-5p, miR-340-5p and miR-483-5p) and 3 DEMirs (miR-335-5p, miR-543 and miR-654-5p) were selected for subsequent gene set enrichment analysis. Targets of every miRNA predicted by two databases, Targetscan and miRDB, were intersected. Consequently, 1612 targets of the 7 UEMirs and 760 targets of the 3 DEMirs were identified (Supplementary Table 1).

### Functional enrichment analysis

**Figure 3.**
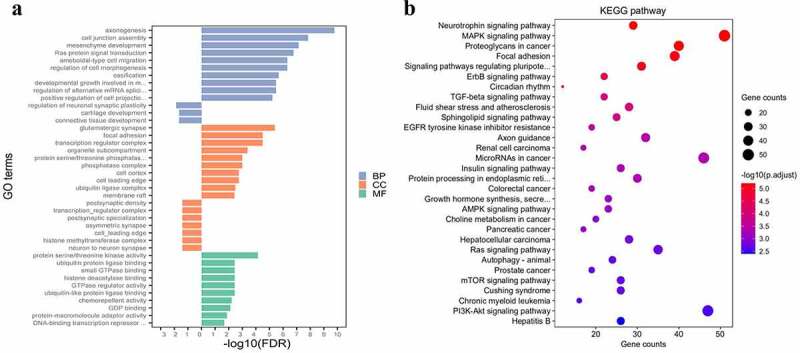
Gene set enrichment analysis. (a) gene ontology analysis of differentially expressed miRNAs. The left part of bar shows enrichment result about DEMirs, the right part shows top 10 enriched categories about UEMirs. (b) KEGG pathway analysis of UEMirs. Top 30 pathway terms were selected according to adjust *p*-value

In the GO analysis, all targets were divided into three categories: biological process (BP), cellular component (CC) and molecular function (MF). For the targets of the UEMirs, 56 BP terms, 13 CC terms and 22 MF terms were identified; the top 10 enriched terms ranked by -log10(FDR) are shown in Figure 3(a). Axonogenesis (GO:0007409, FDR=1.71E-10, counts:89), glutamatergic synapse (GO:0098978, FDR= 4.11E-06, counts: 61) and protein serine/threonine kinase activity (GO:0004674, FDR=7.22E-05, counts:69) were the most highly enriched terms of BP, CC and MF, respectively. For the targets of the DEMirs, 3 BP terms (regulation of neuronal synaptic plasticity, cartilage development and connective tissue development) and 6 CC terms (postsynaptic density, transcription regulator complex, postsynaptic specialization, asymmetric synapse, cell leading edge, histone methyltransferase complex and neuron to neuron synapse) were identified; no MF term were identified.

**Figure 4.**
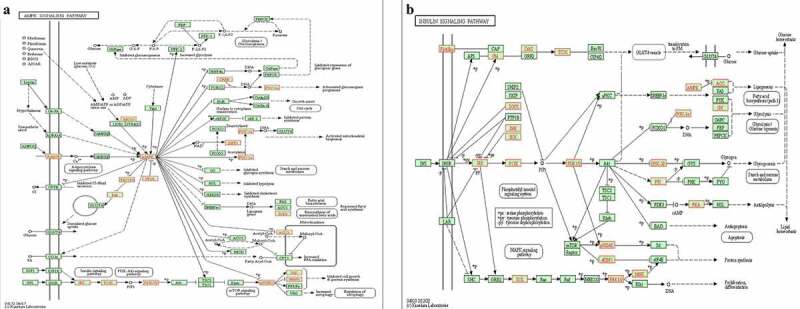
Two interested KEGG pathways. (a) AMPK signaling pathway. (b) insulin signaling pathway. The nodes highlighted in red indicate targets of UEMirs

In the KEGG pathway analysis, the targets of UEMirs were enriched in 68 pathway terms but only one pathway term, oxytocin signaling pathway (hsa04921, FDR=0.02859661, counts:17) was identified in DEMirs (Figure 3(b)). Among the UEMirs-related KEGG pathways, 14 terms (20.6%) were tumor-related. Additionally, pathways such as the AMPK signaling pathway (Figure 4(a)) and insulin signaling pathway (Figure 4(b)) might provide meaningful clues for understanding the pathogenesis of preeclampsia.

## Discussion

Although the universally accepted two-stage theory provides an overview of the pathological development of preeclampsia, the relationship between local deficits in the placenta and systemic impairments remains under debate. Consistent with previous studies [14,15], the current study detected obvious expression of the placental marker PLAP in exosomes from maternal plasma. This suggests that placenta-derived exosomes comprise a considerable amount of the total plasma exosomes and may be an important medium connecting local placental deficits and systemic impairments.

The differential expression analysis of exosomal miRNAs in the current study revealed that sPE had a different plasma exosomal miRNAs profile signature as compared to that of normal pregnant women. Increased expression of some of the differentially expressed exosomal miRNAs in this current study has been reported in the placentas of patients with preeclampsia, impairing the functions of trophoblast cells; these miRNAs include miR-155, miR-215-5p, miR-203 and miR-199a-5p [22–25]. These findings verify that the placenta is an important source of exosomes in maternal circulation. Vascular endothelial cells directly exposed to plasma are important target cells of exosomal miRNAs. Increased expression of the placenta-associated exosomal miR-155 has been reported in patients with preeclampsia and meditates endothelial dysfunction [26]. Additionally, increased expression of miR-203-3p in preeclampsia can cause inflammatory activation by down-regulating anti-inflammatory suppressors of cytokine signaling (SOCS) and the enhancing secretion of pro-inflammatory cytokines in endothelial cells [27]. Interestingly, both miR-199a-5p and miR-200c-3p have been found to suppress the expression of α-Klotho, a senescence-related protein [28,29]. Klotho-deficient mice have shorter life spans and show remarkable aging characteristics such as atherosclerosis, extensive medial calcification and osteoporosis [30]. Treatment with exogenous α-Klotho could protect cells from oxidative stress by enhancing the upregulation of superoxide dismutase 2 (SOD2) and preventing NF-κB translocation, while suppression of α-Klotho can lead to an obvious increase in intracellular oxidative stress and inflammation [31]. The above evidence indicates that UEMirs in sPE may contribute to disease development via mediation of endothelial cell dysfunction.

To further explore the regulatory effects of plasma exosomal miRNAs, gene set enrichment analysis was performed for the target genes of the selected UEMirs and DEMirs. Several selected biological processes and pathways were closely related to preeclampsia, including “cell junction assembly”, “neuron death”, “neurotrophin signaling pathway”, “insulin signaling pathway” and “AMPK signaling pathway”. Interestingly, in both the GO analysis and KEGG pathway analysis, neuron-related gene clusters were highly enriched. Within the “neurotrophin signaling pathway”, several neuron protective factors, such as glial cell-derived neurotrophic factor (GDNF) and oxidation resistance 1 (OXR1) were targets of UEMirs. GDNF, a member of the transforming growth factor-β protein superfamily, has anti-apoptotic actions via up-regulation of the expression of Bcl-2 and Bcl-XL and attenuation of the activity of Caspase-3 in neurons [32]. In addition, GDNF can protect cortical neurons from excitotoxic injury induced by N-methyl-D-aspartate (NMDA) by reducing Ca^2+^ influx and down-regulating the NMDA receptor subunit 1 [33]. Besides, the direct protective effect on neurons, GDNF can also protect the blood-brain barrier by increasing the expression of an important junctional protein, claudin-5, in brain microvascular endothelial cells [34]. The therapeutic effect of GDNF has been validated in central nervous system (CNS) diseases such as Parkinson’s disease and seizures [35,36]. Although the role of GNDF in preeclampsia is still under investigation, we hypothesise that increased expression of exosomal miR-125a-3p may weaken the resistance of the CNS to damage in preeclampsia by regulating the expression of GNDF. OXR1 is another neuron-protective protein identified in the current study. It is known as an anti-oxidative stress molecule because it functions upstream of many proteins related to the stress responses, such as hypoxia-inducible factor-1α, glutathione peroxidase 2 and heme oxygenase-1[37]. Moreover, OXR1 can respond to reactive oxygen species (ROS), the major source of endogenous oxidative stress in cells. When intracellular ROS reaches a critical level, OXR1 is activated and directly binds to peroxiredoxin 2 (Prdx2), a classical anti-oxidative stress molecule, resulting in enhanced anti-oxidant enzymatic activity of Prdx2 [38]. Thus, whether exosomal miR-200c-3p can downregulate the expression of OXR1 in the CNS and then accelerate the development of cerebral injury in preeclampsia deserves further study.

The cell junction between endothelial cells is a factor determining vascular permeability. Suitable regulation of vascular permeability maintains the normal barrier function while allowing necessary molecular exchanges [39]. Dysregulation of the endothelial cell junction is an important pathogenic change in many vascular diseases, including seizures, retinal degenerative disorders and myocardial infarction [40]. Such changes are also related to the development of CNS symptoms in preeclampsia. Increased permeability of the blood-brain barrier was observed in rats with sPE [41]. Similarly, human umbilical vein endothelial cells isolated from pregnant women with preeclampsia showed decreased expression of the junctional proteins cadherin and occludin and increased monolayer permeability [42]. Moreover, significantly increased monolayer permeability was observed in endothelial cells treated with plasma from pregnant women with preeclampsia [43]. The GO analysis in the current study showed that cell junction regulation is one of the most enriched regulatory effects of UEMirs in sPE. Meanwhile, fibronectin 1 and occludin, components of the cell junction, are both targets of miR-200c-3p, a UEMir. Therefore, mediation of the dysregulation of junctions between endothelial cells or the endothelial-matrix junction by exosomal miRNAs may be an important mechanism in preeclampsia.

By controlling nutrient and metabolic homeostasis, the insulin signaling pathway plays a crucial role in regulation of the biological processes of organisms. In endothelial cells, activated phosphatidylinositol-3-kinase (PI3K)/Akt in the insulin signaling pathway phosphorylates endothelial nitric oxide synthase (eNOS), the key enzyme controlling the synthesis of nitric oxide (NO). Then, the phosphorylated eNOS exhibits enhanced catalytic activity and promotes NO production [44]. NO is also an anti-inflammatory molecule that inhibits NF-κB activity and reduces the expression of intercellular adhesion molecule (ICAM) and E-selectin, alleviating endothelial cell activation, which is a process related to higher vascular permeability [45]. Further, activated PI3K/Akt can phosphorylate Forkhead box other 1 (FOXO1) and cause nuclear exclusion of FOXO1, abolishing the transcriptional activation of ICAM1 [46]. Disturbance to the insulin signaling pathway in endothelial cells is present in vascular diseases such as atherosclerosis, chronic hypertension and preeclampsia [47]. As shown in Figure 4(b), several principal molecules (PI3K, insulin receptor substrate and pyruvate dehydrogenase kinase) are targets of UEMirs, consistent with the understanding that insulin resistance contributes to the development of preeclampsia.

The 5ʹ-adenosine monophosphate-activated protein kinase (AMPK) signaling pathway is another important pathway identified in the current study. Key proteins of the pathway are targets of UEMirs: AMPK of miR-125a-3p, and sirtuin1 (SIRT1) and peroxisome proliferator-activated receptor gamma coactivator-1α (PGC1α) of miR-199a-5p. The AMPK/SIRT1/PGC1α axis plays an important role in the AMPK signaling pathway [48]. AMPK, the cellular energy sensor, responds to metabolism stress and rewires metabolism to protect cells from cellular stress [49]. Activated AMPK increases the cellular NAD^+^ level and NAD^+^/NADH ratio, enxhancing the activity of NAD^+^-dependent type III deacetylase, SIRT1 (also known as a classical anti-stress molecule), SOD and eNOS, and diminishing the ROS producer NADPH oxidase (NOX) [50]. Additionally, both AMPK and SIRT1 positively regulate the activity of PGC1α through phosphorylation and deacetylation, respectively [51]. PGC-1α is a central modulator of energy metabolism, promoting the expression of gluconeogenesis genes and fatty acid oxidation-related genes [52]. PGC1α also controls mitochondrial biogenesis via activation of nuclear respiratory factor 1 (NRF1), which regulates the expression of nuclear DNA-encoded mitochondrial proteins [53]. Therefore, dysregulation of AMPK signaling can lead to impairment in metabolism homeostasis and the cellular stress response, which play pivotal roles in the progression of diabetes and cardiovascular diseases [54,55]. However, little is known about the role of AMPK signaling in preeclampsia. Thus, this study provides new insight into the role of exosomal miRNAs in the development of preeclampsia through the AMPK signaling pathway.

There are several limitations of this study that should be noted. First, this study is limited by the small sample size; therefore, the differentially expressed miRNAs warrant further evaluation in larger samples. Second, several possible signalling pathways were screened out. Future studies should validate these findings either *in vivo* or *in vitro*.

## Conclusions

This study has identified different miRNA profiles of exosomes in the circulation of normal pregnant women as compared to women with sPE. In total, 15 UEMirs and 14 DEMirs were identified. GO analysis and KEGG pathway analysis provided information on potential target genes and signalling pathways, which contributes to our understanding of the pathophysiological mechanism of sPE. These findings require validation in larger studies in the future.

## Supplementary Material

Supplemental Material
