# Supplementary material for: Pathogenic mechanisms of preeclampsia with severe features implied by the plasma exosomal mirna profile
Source: Bioengineered. 2021 Dec 9;12(2):9140–9. doi: 10.1080/21655979.2021.1993717 (PMC8810006; doi:10.1080/21655979.2021.1993717)
Supplement: Supplemental Material [file KBIE_A_1993717_SM8811.zip › supplementary patient data.docx]

Clinical data of patientes enrolled

| Group | Age  (y) | Height  (cm) | Weight(kg) | BMI | Gravidity | Parity | Gestational  age(weeks) | Blood pressure  (mmHg) | ALT  (U/L) | AST  (U/L) | PLT  (10^9^/L) | HCT | Uroki-  nase protein | Apgar at 1min | Apgar at 5min | Neona-  tal birth weight  (g) |
| --- | --- | --- | --- | --- | --- | --- | --- | --- | --- | --- | --- | --- | --- | --- | --- | --- |
| sPE1 | 29 | 162 | 72 | 27.43 | 1 | 0 | 35 | 170/130 | 20 | 22 | 131 | 40.5 | 3+ | 7 | 8 | 1800 |
| CON1 | 32 | 166 | 68 | 24.68 | 3 | 1 | 39 | 125/80 | 33 | 21 | 241 | 34.7 | - | 9 | 10 | 3450 |
| CON2 | 29 | 158 | 71 | 28.44 | 1 | 0 | 38 | 120/70 | 35 | 31 | 143 | 35.5 | ± | 9 | 10 | 2750 |
| sPE2 | 31 | 155 | 53 | 22.06 | 3 | 1 | 30 | 160/103 | 27 | 25 | 132 | 33.6 | 3+ | 6 | 7 | 1050 |
| CON3 | 28 | 165 | 62 | 22.77 | 1 | 0 | 40 | 130/65 | 5 | 18 | 227 | 35.9 | - | 9 | 10 | 3250 |
| sPE3 | 26 | 158 | 62 | 24.83 | 1 | 0 | 35 | 160/105 | 760 | 636 | 137 | 39.7 | 3+ | 8 | 9 | 2450 |
