# Supplementary material for: Pathogenic mechanisms of preeclampsia with severe features implied by the plasma exosomal mirna profile
Source: Bioengineered. 2021 Dec 9;12(2):9140–9. doi: 10.1080/21655979.2021.1993717 (PMC8810006; doi:10.1080/21655979.2021.1993717)
Supplement: Supplemental Material [file KBIE_A_1993717_SM8811.zip › SupplementaryTable.docx]

**Supplementary Table**

Targets of differentially expressed plasma exosomal miRNAs

| UEMirs | targets |
| --- | --- |
| miR-199a-5p | ZNF776,ZNF439,ZNF544,ZNF709,ZNF584,ZNF547,MAP3K11,LIN7C,DDR1,BCAM,SHOC2,HAPLN1,ZFP2,TST,MYRF,CELSR1,GPR63,CDCA7L,NSG1,RAD23B,ZBTB42,CRYBG3,RBM47,PDPN,HMCN1,MGAT4B,MAB21L1,ARHGAP21,TSPAN6,ECE1,SULF1,GCNT2,AKAP1,FAM222B,ALS2,GPR89B,SLC24A3,TGFB2,ZNF329,EPB41L1,VPS26A,FLRT3,CCDC43,GJA5,PODXL,FZD6,ARF6,PPP1R2,ARHGAP12,TAF9B,GPR89A,RASSF2,UBL3,ABHD17C,ASRGL1,SLC25A23,CCNJ,SUN1,SOS2,RAB9B,8-Mar,NINL,CACUL1,ZNF579,BICC1,NAA40,RGMA,GRB10,CCNL1,GNG5,RALGAPA1,ZFYVE27,SNAI1,DPP8,NTNG1,AP1G1,GPRC5A,HSPA5,CACNB2,PPP1R9A,MYEF2,CCDC120,FZD4,SRRM1,PPARGC1A,CLTC,CSGALNACT1,WNT2,MICAL3,GRIP1,IPO8,ABCC1,SACS,JUNB,ETS1,MUC21,PAX3,SORCS3,TMEM63B,7-Mar,OSR1,RNF11,ZNF516,NPAS2,PKN2,KPNA4,SLC9A8,HSPA12A,CAPRIN1,RLIM,EXOC8,LARP4,TSPAN3,KIAA1109,RANBP2,NAALADL2,PNPLA6,UHMK1,BTBD3,TSPAN5,CSDC2,ITGA8,TBC1D8,FER,RBPMS,UBAP1,PAN3,TBC1D14,DUSP14,PPFIBP1,EIF5B,RAB21,PHACTR4,WDR44,CREBRF,ANK3,ITGA3,CHN2,MCFD2,MINK1,GPD2,ACVR1B,GSK3B,TTC9,TAB3,FBXO30,NLK,BROX,ATP13A2,PAXBP1,CCDC88C,PDE4D,TOX3,STK4,GPR180,EMC10,SIRT1,MARK4,KLHL29,SUCO,BEND3,ZNF652,MIER3,ADD3,EMC7,CELF2,NFIL3,PARP12,MAP4K3,CDKN1C,KLHL23,CECR2,FUT9,ATXN7,PMP22,ERLIN1,RNF38,WDTC1,XYLT1,RASSF3,DNAJB5,DYRK1A,NCSTN,RUNX1T1,DENND6A,HOXA7,COL5A3,SEMA3F,TRAF3,ATG4D,TMEM245,CNN1,PLXNA2,JPH3,ZBTB18,TET2,APPBP2,ACTG1,TAOK1,USP27X,MATN2,SET,GIT1,PREPL,USP37,CLK2,SMARCD1,RORB,HIF1A,DDX3Y,IKBKB,BTRC,SLC24A4,TMEM135,SMARCAD1,CLIP1,LAMC1,SLC35E1,FBXO33,SRGAP3,RFX3,STAG1,CLCN3,ZCCHC2,MN1,EPHA7,CLOCK,PI4KA,UBE2Q1,SORL1,OSTM1,NOTUM,PPP6C,MYH9,ZNF148,SLAMF8,AUTS2,ONECUT2,SLC25A37,ZFP91,ARHGEF5,KANK2,SOX4,RRAGC,SNN,AFTPH,SLC24A2,AGO1,CAV1,EXOSC3,ERBB4,GBP1,PCYOX1,SH3PXD2A,PLEKHH1,MGAT3,RPS6KA5,SLC35A3,PDE7A,MPP5,STON2,FAM126B,ZNF704,BTBD9,PBRM1,ABCA1,NFE2L1,RIMS1,E2F3,ATXN3,ARIH2,HOXB6,NAA15,USP46,ATP1A2,REEP2,ARHGAP19,WNK3,KIT,KIAA0355,CBL,POGK,COL8A1,GANAB,ATP2B2,ARID2,BCL7A,NFYA,SP1,CEP85L,ACVR2B,PURA |
| miR-200c-3p | VASH2,HIPK3,MAP2,ERRFI1,ZEB1,NR5A2,ZEB2,RECK,SLIT2,FAM8A1,AP1S2,SEC23A,PTPN21,DNAJC3,QKI,CCNJ,PCMTD1,CFL2,GPM6A,ARHGAP6,TFAP2A,BAP1,RAB11FIP2,MSN,FBXW7,TRIM33,CNOT6,RPS6KB1,NOVA2,WASF3,ELL2,ATXN1,MIEF1,MBNL3,CSNK1G3,KHDRBS1,ELMOD2,MCFD2,CRKL,PPP4R2,MGAT2,NFIA,FEZ2,PRDM16,LRP1B,PPP2R5E,PTPN14,OSTM1,CDK17,FRMD6,PIK3CA,CCDC177,ARIH1,ATP11C,RTF1,PPM1F,RAP2C,MMD,CHN2,PHF21B,SULF1,ARL2BP,ADIPOR2,ZNF532,PHACTR3,RASA2,ZNF711,SEMA6D,VLDLR,CBL,EPS8,NANOS1,TMOD3,COL4A3BP,KDELC1,RND3,KDR,NCOA2,HMBOX1,DUSP1,C16orf72,EGLN1,CLASP1,ZFAND6,FOXG1,MBOAT2,PHTF2,NBR1,ZNF131,AFF3,ARL5A,IMMP2L,MAP3K1,SGIP1,GOLGA7,DLC1,XKR8,FN1,SESN1,JUN,GPR158,DGKH,SYDE1,ZC3H6,PRKG1,SLC1A2,RANBP9,GOLGA1,LOX,GIT2,PRKACB,CDH20,NTF3,B3GNT2,PSAT1,REEP1,MED13,TBK1,TBC1D12,VEGFA,RUSC2,SFXN1,HS2ST1,RAB21,DESI1,FOXF1,TMEM17,FSCN1,PPP1R18,RNF2,THAP1,CLIC4,CECR2,HOOK1,CHRDL1,DTNA,DNAJB9,NR3C1,CSMD3,PPFIA1,PI4K2B,VASH1,PDS5B,SLK,SPAG9,AMFR,ELAVL2,LBR,ZYG11B,CLIP1,WDR82,SERPINI1,RASSF8,JAZF1,TOB1,EVI5,POLK,TRAPPC8,PUM2,CDYL,YWHAG,SLC6A11,TCAIM,PKD1,FBXO30,DNMT3B,FBXO33,OCLN,VAT1L,SCAMP1,GABBR2,ADAMTS3,NEDD1,LRRC8A,NRBP1,S100PBP,USP25,CAMSAP2,PPP1R9B,PMAIP1,TSC22D1,PSIP1,MFAP5,SLC4A7,NOG,SLC6A1,MIB1,DCBLD2,DENND5B,CCNYL1,CKAP4,STRN,6-Mar,TMEFF2,TLN2,HSPA13,SLC14A1,PAG1,PIKFYVE,COPS8,USP27X,PTPN12,MARCKS,AGFG1,CBX4,DIXDC1,NOVA1,DGKA,ZNF217,HS3ST1,GXYLT1,INTS8,RIMS2,GLI3,YPEL2,HDHD2,TIMP2,USP6NL,ERG,OSBPL11,MATR3,ATL2,MAPRE1,DNAJB5,GNAQ,MAP4K3,CNEP1R1,RIPK2,ZBTB38,FOXN2,ETS1,ELK3,CEP41,CNKSR3,ULK2,ZFPM2,ZMAT3,RDH10,CCDC82,SCD,XKR4,ZCCHC24,SMARCD1,ZSWIM4,NCS1,PLCL1,CYTH1,TRHDE,BCL11B,FLII,EIF2B5,FERMT2,SERINC1,RANBP10,RBFOX3,HMGB3,GJC1,CORO1C,TAF12,TSC22D2,CPED1,WWC3,BNC2,PKIA,RAP1B,PTHLH,C11orf87,CLASP2,KLF4,SCRT2,GPATCH8,SGCE,SLITRK1,WASF1,FLI1,FAM118B,SIX1,PI4KB,SLC39A14,NUDT4,PDIK1L,CDYL2,FIGN,HIPK1,JKAMP,SDC2,DACH1,PPP1R10,SLC35E2B,SYVN1,DZIP1,SOX2,GLCCI1,BDP1,HS3ST3A1,KIAA0355,ARHGAP20,APOO,SLC24A4,FSTL1,CASZ1,CTDSPL2,CHSY1,KANK2,NPC1,ARID4B,BICC1,MYZAP,SEMA3F,CASR,HNRNPD,MTF2,UBE2W,MYB,IER5,CRTAP,SECISBP2L,PRDM1,GATA4,NYAP1,SCN8A,NRG1,ACVR1C,MTSS1L,SBSPON,KLF6,EFNB2,KCND2,MSL2,FRMD4B,OXR1,CDH11,BPTF,RSPRY1,NDN,THSD7A,PHF6,ARHGEF17,ETV5,ANLN,BAG5,UBQLN1,LAMC1,XKR6,FYN,PPP1CB,RIMKLB,HOXA5,GABPA,KANK1,LMO7,PCSK2,SH3GL1,CNN3,CNTFR,LCA5,RAPGEF2,IKBKB,SPTSSA,NFIB,LARP1B,UBE2D1,GTF2E1,SBF1,SIX3,UBA6,C6orf120,SLC16A2,GNAI3,EFNA1,PAK6,ZNF697,CDR2,TMEM164,MXD4,GAB1,ICK,TBC1D22B,GRAP2,NCOA7,FBXO22,UBE2I,MIER3,CACUL1,SOX1,PRKAR1A,FUBP3,TIAL1,MEX3B,GPR173,IQCJ-SCHIP1,ZBTB8A,TMEM170B,EIF5B,PAIP2,RBFOX1,SNAP25,CERS6,GMFB,NFYA,HNF1B,8-Mar,ZDHHC17,WDR91,CALU,RSRC2,LPAR1,RHOT1,N4BP2,RNF38,RBM26,CDC73,PLS3,ADCY2,TMEM170A,DCAF17,PALM2,SRP72,ARGLU1,TMEM33,BAG6,TLL2,DOCK4,FAM81A,MXD3,CNST,PPP2CA,CTNND2,ORMDL3,SRF,STARD13,SSR3,FUT4,LFNG,UBE2V1,FHOD1,SYT1,ZNF292,TBX5,MMD2,RAC1,TMEM189-UBE2V1,CHST2,NLGN4X,BTF3L4,NPTX1,MTFR1,RLF,CNTN4,C6orf62,DIRAS2,RBM12B,UGCG,PHLDB1,SRSF1,CYP1B1,PPP2R2C,SLC35F4,ASF1A,INPP4A,SNAI2,LPIN1,TXLNG,PCNP,PIP4K2B,NCOA3,ARHGAP19,FXR2,PLCG1,PHF21A,SNAPC1,PARD6B,RELN,KCTD15,PSPH,PIN1,CHD2,TMCC1,ITPR1,CHST9,PRKAR2B,MYCN,ARIH2,CSRNP3,NEGR1,PTBP1,LRP4,BASP1,SCOC,GFI1,TMEM229B,ANKRD28,WDR45B,FGD1,FMR1,OSR1,FRS2,TP53INP1,GATA2,LRRTM3,FUBP1,CDK2,ATMIN,KLHL14,RALGPS2,PAN3,CCSER1,CASC4,ITSN1,PPP1R12B,TENM1,BHLHE41,KIF13A,PIGM,CKLF,GLIS2,TARDBP,CEBPD,NEK9,SMARCAD1,ERI1,TUBB3,PDPK1,VTI1A,PIK3CB,HLF,AMOTL2,KBTBD6,ARL6IP6,SNX30,CDH6,ARHGEF3,ZBTB5,ZMYM4,HNRNPU,LMAN1,KLF10,SCHIP1,TTC5,EIF2S1,CALHM1,TTC33,ADD3,PTPN13,ABI2,DR1,DNMT3A,RHOA |
| miR-483-5p | RUSC1,CT62,IQCE,STK40,ALCAM,HR,NUDT8,RNF165,CUTA,MYOM2,FN3K,KATNAL1,LMO1,CDK15,DDI2,MSC,SRSF4,KIF4A,MAPK3,STX6,TSPYL5,CLCN3,CBS,TMCC2,FAM160B2,CACYBP,BBS5,UGT3A1,RPL31,TIMP2,ACBD6,WDR92,DLG5,ZNF584,GFRA4,GPT2,TNN,HGSNAT,ZNF417,RAX2,IQSEC2,SMG6,PAX2 |
| miR-340-5p | MAP3K2,PNO1,ZC3H12B,ATF1,DYNC1I2,HS2ST1,DCUN1D1,CAPN10,RAP1A,UBE2D1,CXADR,ZFPM2,NR2C1,XRN1,LPP,PAIP1,VGLL3,ARG1,MSRB3,PLEKHA3,FZD3,UGDH,SMIM15,SNX3,NOG,TOX2,EDNRB,MBNL3,HOXB2,CDC42SE2,EVI2B,SLC30A6,TCF21,IGFBP3,SUMO2,CCNG2,KCNA4,RBM7,TCP11L2,FRMD6,SNX6,CHIC1,NFE2L2,KRR1,RGS4,MED17,SGCE,ONECUT2,RAB27B,FDX1,RAB11A,C5orf47,DDX5,NHLH2,KCNS3,PRKRA,ACTC1,ATXN7L2,FAM19A1,PLAT,LHX1,AHR,RORC,C1QTNF3,IFNG,ZNF292,PTP4A1,SLC25A32,BMPR1A,TRIP4,NT5C1B-RDH14,POPDC3,SVIP,RP2,CEPT1,VHL,LEPROT,VPS54,RANGRF,OSTM1,EIF3J,SIVA1,HNRNPH3,MED28,KIT,SAMD12,APLN,KIAA0895,ZNF513,HSD11B1,RHOA,MZT1,VMP1,RNH1,PLA2G16,ZBTB37,NUP88,ZNF664,RWDD2A,C1orf52,DLX3,B2M,TMEM67,NCK2,ARHGAP29,SPRY3,CD69,TVP23B,TMEM170B,COX17,WNT11,LDB2,ZDHHC20,KRCC1,RCN2,CSMD3,MAL,DNAJC19,FHL2,C3orf33,ZNF777,H3F3A,ESYT3,BTBD10,C1orf43,AK3,SLC25A30,TMEM216,ANO6,HERPUD1,NFE2L3,THG1L,RNF7,ALAS1,DEK,IRF9,ATP12A,SPCS1,SKP1,UBE2E3,ICAM5,MLF1,C16orf74,TM9SF2,LUC7L |
| miR-203a-3p | LPP,CAMTA1,ADPGK,PRPS2,SMC5,TMEM69,CADM2,ALG10B,GABRA1,SLC4A4,GRHL3,TMEM100,FAM126B,TTC39A,NSG1,ID4,C4orf33,SOSTDC1,SH3BGR,PCBP2,DKK2,HNRNPL,DCX,GPATCH1,TAOK1,RAP2A,CREB1,PRKAB1,LRRCC1,PHLDA1,TDRD6,ADK,TEDDM1,SMAD9,RFX6,HNRNPUL2,TSC22D2,GLS,RHOJ,NCL,EBF3,APPL1,KCNN3,COPS7B,KIF2A,SMURF2,RHOQ,HOOK3,SCGB2A1,HAPLN1,IL24,ZNF281,DUS4L,RNF34,SEC62,CCNG1,KCNJ15,PARM1,MBNL3,TNC,IMPA1,MAP3K5,SLC30A6,YAF2,CLVS2,ZFHX4,TMEM248,DUSP5,CUL1,DOCK10,CSN2,ANTXR2,ATF2,PRKG1,NAA30,ZMYM2,ABCE1,MAP3K2,CITED2,TBCEL,PAQR3,GPR85,C7orf25,RTKN2,SRSF1,MAP4K3,SLC17A6,DGKH,KLHL14,XKR4,PRDM10,DNAJC21,PITX2,CREBZF,TWF1,OVOL1,CNNM3,ZNF148,KAT6B,AFAP1L2,MAPK8,EGR3,LCOR,ZNF197,PPP1R12A,KRT1,BMI1,LNX2,NEDD4L,KRT85,SGTB,C11orf91,PRKCB,KLHL15,BTAF1,CREBRF,PAPSS2,TADA1,SGMS2,PEX5L,AFF4,YWHAQ,MBNL1,MATR3,ARHGAP42,TMOD2,SESTD1,FOXN2,EIF5A2,WRNIP1,ARHGAP12,COMMD3-BMI1,TLL2,KCTD9,NIPAL2,SHH,SEMA5A,SOCS6,TCF4,GXYLT1,AHR,DCP2,CDH10,CLOCK,RAB10,GMFB,UBR1,ZC4H2,UPF2,USP8,TADA2B,SIX4,SCN1A,SRA1,SPARC,SNAI2,MBNL2,PLD1,RAB27B,MCTP1,GUCY1A2,AP1G1,OPA1,NFYA,SPIRE1,GPC4,IRS2,KRT35,ZNF292,PDE4D,VSNL1,PRICKLE2,DPM1,RBM47,TNFSF15,MSI2,SLC39A9,TCF12,ZBTB20,AAK1,CTDSPL2,DR1,NEDD9,CHD9,TRIM71,NFIB,TIAL1,UGCG,SLC7A14,NETO2,NPR3,KHDRBS1,HDX,UBN2,PDGFD,DLX5,PTP4A1,FAM135B,LASP1,CAB39,DCUN1D4,ACVR2A,BMPR1B,DIP2B |
| miR-215-5p | NIPAL1,BHLHE22,LPAR4,ZEB2,EREG,WDR44,WNK1,RAB2A,DYRK3,OSBPL10,FRMD4B,MTMR4,NKAIN2,ARFGEF1,ZBTB34,PDP1,SRSF6,CCNT2,CXCR5,RB1,MSN,PKP4,ZFHX3,PHTF2,MIPOL1,DDX50,TSHZ2,XIAP,CTCF,RAD54B,NIPBL,MYLK,IGDCC3,GABPB2,GALNTL6,ALCAM,GDF11,FAXC,SERINC4,C4orf46,CHD7,FOXN1,EMC7,KPNA4,RUNX1,GPR22,SH3RF3 |
| miR-125b-5p | SH3TC2,BMF,STARD13,ZNF704,GCNT1,ARID3B,IER3IP1,BLZF1,KLF13,CGN,NUP210,NIPAL4,IRF4,RORA,DOCK3,KCNS3,TMEM135,NBEAL2,BAK1,ACHE,KCNK10,PI4K2B,CYP24A1,KCTD15,ZSWIM6,NPL,TTPA,ETS1,SBNO1,KIAA1841,OSBPL9,LIPA,SSTR3,MTF1,DUS1L,VPS4B,CDH5,NECAB3,ENPEP,SLC39A9,ATXN1,SEL1L,SEMA4B,RAPGEF5,KCNIP3,BAP1,UCK2,C19orf38,SLC35A4,MAPRE2,MORC2,ATP10D,RBM20,ABHD6,UBN1,DHX33,PHF20,RASGRF2,CCNJ,TMEM120B,XKRX,KIAA1522,TMEM168,VTCN1,OLFML2A,SGPL1,ZSWIM5,SARM1,USP2,TRIM71,SLC7A1,PRDM1,ACER2,NCAN,TAZ,PPME1,WARS,INO80D,DIS3L2,ALPK3,SLC25A15,SLC25A35,ABHD3,RABEP2,SLITRK6,ZFYVE1,TOR2A,DIRAS1,UBE2R2,DPH2,NRXN1,TMEM161B,RASGRF1,INTS7,SYVN1,TSEN54,ULK3,PAFAH1B1,ENPP1,RHOQ,ANKRD33B,HIF1AN,TNFSF4,FAM169B,MFHAS1,CDC42BPG,TRIAP1,DUSP6,RAB3D,EVA1A,MAP3K11,LIN28A,CRB2,MFSD9,ESRRA,SEMA4F,ITGA8,LFNG,ZNRF3,TLE3,DRAM2,SUV39H1,NCOR2,ZBTB37,TSNARE1,IL6R,PHACTR3,KCNA1,ABTB1,CBFB,FBXW4,LIN28B,SCARB1,SLC46A3,TMTC2,HAPLN1,FRMD5,PRRC1,MYT1,SLC38A9,NR6A1,NEU1,SEMA4C,ZSWIM4,RFXANK,UBE2G1,MAP3K9,IL16,KLC2,KIAA0319L,CDR2L,MAMDC2,SCLY,LRP4,RFX3,TOMM40,NT5DC1,HCN3,GALNT14,IST1,SH3BP5L,E2F2,PRSS35,BNIP2,CHTF8,SAMD10,PPP2R5C,TTC7A,RBM7,TMEM132E,CACNB3,PMM2,BAG4,LBH,MKNK2,EIF4EBP1,ORC2,RPS6KA1,PCTP,EIF1AD,SLC26A6,TRAF6,DAAM1,C6orf47,ANKRD50,LGI2,RAP1A,CDC42SE1,RASSF3,TBC1D16,GRB10,KCTD21,MAP3K10,MAPK12,PLEKHA8,USP38,MAN1B1,TNFAIP3,HINFP,SLC6A17,UBR7,TAF9B,PDE7A,VCPIP1,QSOX2,FAM118A,SCN2B,SERTAD3,CEP85,SEMA4D,VDR,RUFY3,TBC1D1,RREB1,DENND6A,GOLGA5,MSRB3,NCKAP5L,PLEKHM3,CDK19,BTG2,SUN1,MBOAT2,C1orf210,LRRC10B,EIF2B5,GJC1,GGA2,SNX18,LCLAT1,TBX4,ZBTB7A,LRRC8B,KHNYN,SRF,RBAK,SLC16A6,PPP1R37,SULT4A1,IGSF11,PDK3,ZNF652,EBF4,SLC4A4,ZNF385A,ASIC1,ZKSCAN5,AIFM1,RASGEF1A,VPS36,RYBP,LPAR4,ZDHHC9,PODXL,FNDC3B,ZBTB34,TXNRD1,NIN,ELOVL4,CSRNP1,OAZ2,DPP9,GPR107,IER2,ELOVL6,PCGF6,SZRD1,SCUBE3,MTMR3,LRFN2,ENTPD4,KCNH7,HOXD9,MAP2K7,ATXN3,ESYT1,RUSC2,C7orf26,ST8SIA4,SCRT2,CNNM1,ANPEP,VPS37B,FBXO45,BRWD1,MSI1,EAF1,RFX5,TDG,PLXNA1,RAB6B,DNAJC14,TEF,ZC3H7B,ERBB3,STAT3,AKT1S1,DNAJB5,ABCC5,E2F3,ZFP62,PRKAA2,FOXD2,ATXN7,AKAP13,CASP2,NCLN,TYSND1,SMG5,NAIF1,PPP2CA,FAM78A,PKP4,LYPD6,STXBP5L,CAMSAP3,RAPGEFL1,ELAVL4,ZNF76,USP46,FGFR2,SPSB4,HDDC3,NFIB,RNF217,ESRRG,C19orf54,ANO6,MBD1,GPC6,FAT4,SCN4B,RALGPS2,SIRT7,PHYHIP,SH3BP4,SAMD14,TET2,CORO2A,KLHL6,PPM1H,ATL2,GTPBP2,TP53INP1,TRIB2,TGFBR1,APC,TSPAN12,CCNJL,UBTD1,NEDD9,MGAT4A,SMG1,LPCAT4,GPR153,TMEM123,TGOLN2,ADAM9,ANO3,ARSI,GLB1L2,C10orf105,EDEM1,BCAT1,FAM131B,TIMM17B,C15orf39,ZNF792,QKI,SORCS2,MLX,WDR1,MEGF9,ANKRD13B,PACS2,MIB1,CACNB1,PROX1,CBX7,PTAR1,KCNJ12,THEMIS2,CGREF1,DUSP3,KIF18B,AREL1,RHOT2,ITGA9,PCSK7,SLC4A10,CYTH1,RBM24,KDM4B,DAZAP2,ADAMTS14,ELMSAN1,MTUS1,GAB2,ARID3A,SRRM3,ZNF460,KLHL31,KPNA6,SLC4A8,COPS7B,FLOT2,ANTXR2,LIFR,BCL2L2,ST6GALNAC6,PPP1R12B,USP37,LOXL1,YOD1,CCDC71L,GGT7,THY1,TRPS1,PEAK1,BCL2,HOXD1,VWC2,HK2,TACC2,RIT1,FAM174B,LCOR,NUMBL,STX6,YWHAG,IMPAD1,KCNB1,LURAP1L,BDH1,RNF44,CYB5D1,KLHL24,CORO2B,ZNF699,COPZ1,GPC1,ACACB,PPT1,NRM,KCNH4,RNF121,ASXL3,SLC24A2,SOCS4,BCL2L12,PAN2,ARCN1,OGFR,FAM107B,GOPC,ST6GAL1,RHOBTB2,GALNT7,SWSAP1,BRPF1,AGGF1,ELL,SNX27,TMEM198,AMER2,OVOL1,CECR2,PGAP3,ATP13A3,SORT1,COL4A3,STC1,SLC35C1,TLK2,DCP1A,TMCC2,UBE2W,TSTA3,DPF2,GRSF1,ABL2,STOX2,GDNF,ENTPD1,MEIS2,ZNF304,ACVR1C,H6PD,SH2B3,DDX42,FAM129B,PTPN18,ZNF236,PGP,ATG4D,ACSL6,MEGF8,ITGA1,MLF2,PSTPIP2,ZC3H12B,RNF144B,OPALIN,TRAPPC6B,NDRG3,ADAM11,TNFRSF1B,ZNF281,DUSP7,BMPR1B |
| DEMirs |  |
| miR-335-5p | CASP7,SEPHS1,ZRANB1,NRXN1,POU2F3,HOXD8,DAAM1,NXPH2,VAPA,ARGLU1,PSD3,HAND1,UBE2G1,RPRM,CNOT7,PRKAA2,CALU,FBXO28,FAM131B,GLYR1,SNIP1,SMARCA2,EIF5A2,PGM3,KAT7,CCNF,NCKAP5,POU5F1,F13A1,SECISBP2L,SORCS1,NAA25,ARHGAP18,ZMPSTE24,KDM4C,CAMKK2,RASA1,GJA5,APTX,SREK1IP1,MED21,FKBP1B,MAX,KAZALD1,ZDHHC8,SLC45A3,MED6,KDSR,CCR3,ROCK1,MAPK10,KLHL15,PPP6C,CORO2B,KLHL28,PRDM10,RNF141,NR4A3,PIAS4,IL17RD,VAV2,EFNB1,TRIM5,ZEB2,KPNA6,MAT2B,EVI5,SUCNR1,KDELR1,SPTSSA,STIM2,SGMS2,ADCY3,EEF2K,PLEKHO1,GDE1,ZNF621,TFDP2,STRN3,ARPC5L,CTDSPL2,CLOCK,CPZ,CRIM1 |
| miR-654-5p | PELI3,DIRAS1,EPHA8,GLP2R,AGO1,ITM2C,HAX1,LHX8,MTSS1,LSM12,STK38,GEM,COTL1,TCOF1,ZGPAT,TSPAN9,MGLL,MYOCD,ZFHX2,PARVA,ZBTB4,LENEP,KIAA1324L,STMN2,RANBP10,IRS1,BCL7A,RAB11FIP1,CTDSP1,JPH4,TMEM11,SLC41A2,KIF17,AMIGO1,MEF2D,SUPT16H,NOVA2,RAB1B,WDR82,DDX17,CD2,LMOD1,TGM2,TSPAN3,PTPRN,SLC6A8,LRTOMT,ALPK3,CYB5R3,DDR1,PRX,CACNA1E,HLA-DQB2,XIRP1,MOCS1,EDAR,PPP1R1B,CABP2,KCTD15,MRAS,HOOK3,DBH,RBM43,ZNF275,MLEC,GNAS,ATG2A,ENOSF1,SLC6A17,PTPN23,PITPNM2,GUCA2B,SLC25A42,YWHAH,SMG5,NFATC3,RPTOR,CPLX4,DCN,PRR14L,BAZ1B,TMEM178B,INO80E,NREP,HLA-G,HDAC7,NCS1,IL17REL,STK32A,ZMIZ1,TNRC18,L1CAM,NUDCD3,PBX3,PCDH11X,SPATA16,LHPP,NFIA,WNT11,MSL3,ELFN2,FBXO46,C9orf66,GH2,HPCAL4,HNF4A,ANXA11,FLT4,SPECC1,VDR,TOM1L2,MSI1,ERGIC1,PPP1CA,TRPC4AP,FIBCD1,MLF2,SRM,ASIC1,ABCB8,SIRT6,GH1,MDGA1,CAMK2A,CYP4A11,IL4R,CSHL1,GNG13,SETD1A,DMRT2,CSH1,LDB1,ZHX3,APH1A,TRIM67,STC1,LRCH4,BMP1,PGPEP1,OBP2B,ARHGAP27,TMEM129,C2CD3,SH3BP2,FGF19,WBP1,PAX8,CBX7,TGFB1,CNIH2,INPP5B,OSBPL10,GTF3C4,RFXAP,SLC25A47,TMEM150A,ETV6,ZFYVE1,RSPO4,GDF5,ZBTB7A,SYNGAP1,FBXL20,KIRREL2,MGAT3,NOTCH4,IRAK3,PEBP4,FCHSD1,MAP3K1,KIF21B,LIF,CNOT4,SYNGR1,WFDC8,ZNF709,NFAM1,CRAT,FOXP4,NKAIN4,TMEM63C,ZFYVE28,CD276,PALM,WAS,HPS1,FADS6,CEP170B,ACACB,TNRC6A,SAMD14,NELFB,ANKRD52,RASD2,DNAJC24,POU2F1,ATP11A,TMEM239,GPR17,SNURF,CRX,ZNF93,COL4A3,GDPD4,TSPAN17,ZNF418,FOSL1,APCDD1L,DERL3,DGKZ,SORBS1,AK4,SOST,KCTD11,FKBP10,PYGB,DGKE,SHISA7,MAFG,NPHP4,ZNF629,STK35,TMEM140,CDC6,PIK3R6,TMEM120B,N4BP1,PPT2,SEZ6,ITPRIP,HOXA11,SCGB1A1,TRIM9,ADAMTS7,CRTC1,SMARCC2,LMX1B,GLRX3,GRHL2,CNNM3,LDB3,PEX26,KLF7,TYK2,DHX58,STX1B,S100A1,CLN6,ATP13A1,PLEKHM1,ING1,DMRTA2,ZBTB3,SYNDIG1L,LCN8,PPIA,MTHFSD,ELN,ME2,CSNK2A2,ITGA3,SLC43A2,CLCN6,MRPS16,LYSMD1,CRABP2,C5orf60,ELMO1,HCFC1,LFNG,RALGPS1,GLCCI1,SETBP1,UNC5C,CAMKK1,FAM53B,CNNM4,ICMT,CHD3,TEX261,PPM1G,KIF3C,CELF5,OS9,GRIN1,TMCC3,CKB,DAGLA,PHF8,GSDMD,MEX3A,IKZF3,SH3D19,MALL,TTYH3,PVR,SCLY,ELOVL1,RCOR2,DYNLL2,BTNL3,CLEC18B,CHST11,B4GALT4,TRIM5,AP3S1,ZNF124,C1QL1,CAPN12,TLN2,CGN,C16orf72,TMEM241,OR10W1,RAD54L2,SEPT9,NKAIN1,LRRC14,SH2D1B,ARC,SUB1,TRIM46,ZNF516,BMP8A,NEDD4L,GTF2E1,MRPL11,ZDHHC3,SLC26A1,GLYR1,HYOU1,PRDM6,RGPD1,OPA3,SYT11,LY6G5C,AP3S2,PFKFB2,ST8SIA2,SNX33,TEX28,PIM3,ATP6V0C,SV2C,CASKIN2,ADD1,GALE,SYPL2,AMER3,MASP2,NODAL,ACE,TMEM132E,MMEL1,MED8,SPATA33,BCL9L,TAS1R3,GPR132,SOX10,NXF1,MUC12,PRKAR2A,RAVER1,NFE2L1,NACC2,CACNA2D2,C1orf226,RNPEPL1,CABP4,DNAJB5,PDCD1,AGXT,C16orf58,NUDT8,PNMA5,CLEC16A,RNF41,ATP6V1G2,RBBP8NL,COL11A2,CD7,CTCF,SH2B3,RNF4,EFNB1,ISY1-RAB43,ADAMTS17,HOXB9,USP37,CACNB2,ZNF385A,AMPD2,KDM6B,PLEKHA6,TNFRSF21,ZMYM3,KCNJ11,BLOC1S5,ADARB2,DNAJA4,MYO1C,SKI,SMIM14,EVC,SLC9A3R2,TRIM26,RECQL5,TSPAN11,GREB1,SH3BP4,THEM6,PROP1,USP13,TIMP2,NPAP1,RAMP2,RPL28,EXPH5,KCNAB3,FGF18,AVPR1B,KRTAP4-7,PKP2,EHMT2,DPYSL2,TNFSF15,CHRNA4,PIP5K1C,ANO9,SMARCC1,CAPN14,ZNF333,SLC37A1,ATF7IP2,ZNF578,CNPY4,RBM38,ZNF587,MUC6,PCYT2,UNC5CL,PEA15,TOX2,RASL10B,ANKS1A,FZR1,GATAD2B,HRH3,GDI1,ZNF490,HSPB1,GNPAT,SEPT3,PAQR8,EFHD2,PARN,KCNJ10,MRPL49,AFF3,SCN2B,HIF3A,ZNF319,SLC7A1,ARAF,BCAS3,HOXB6,BBC3,CLEC11A,FNTB,AFAP1,ZNF395,TSNARE1,RANBP3,APLN,TAB1,CBX5,UBXN10,PPM1H,CHURC1-FNTB,PTRH1,NAT8L,NICN1,CALM3,CD3EAP,LIMD2,TBX3,SLC25A34,WNK4,LMO1,ALOX15B,SCIMP,S100PBP,NRSN2,SAE1,KCNJ6,SNED1,NACC1,SMPD3,ZNF736,MRPL10,COBL,EMILIN3,ADAP2,TMEM151B,TMCO4,GPC1,KCTD13,RTP1,MRPL4,ZNF710,AR,KBTBD12,RAPGEF5,APOL1,PCYOX1L |
| miR-543 | FBXO34,TBC1D1,GPCPD1,KLHL5,ZBTB43,IL1A,SLC38A4,STT3B,FMNL2,TNFSF11,LMO1,MTF2,DYNC1LI2,FIGN,LRRC8D,WASL,TCERG1,RBM47,UBE2W,CYB5R4,ABHD13,ADAM9,OSBPL3,SS18L1,CTDSPL,ANKRD13C,PCSK1,EPS8,METAP1,RAB30,HS3ST3A1,BIRC6,MIA2,PDE5A,SIRT1,CDH2,FAM160A2,STRBP,PLA2G4A,GPBP1,GABRA1,NMT2,ING1,B3GALT1,HECA,SERPINI1,TCF7L2,CASK,ZDHHC3,FAM117A,ID4,C16orf95,ZNF704,TWIST1,REPS2,CTTNBP2NL,FAM160A1,EIF4A2,ACVR1C,SLC10A7,DLX1,C5orf30,SH3GLB1,NANP,IQCJ-SCHIP1,RNF13,KLHDC10,KIN,METAP2,PDAP1,ING5,AKAP5,RAB11A,CCAR1,PPP2R2C,MARC2,RABL3,RP2,EIF1,FKBP4,SIX2,RABGEF1,NCK2,KRAS,ERLIN2,MAF,TXLNB,PCBP1,TRIM39,RNF2,FOXP1,DNAJC10,PAWR,PBX3,BTBD3,CNNM2,AK3,AASDHPPT,MSL2,C12orf56,MLF1,NEGR1,SCOC,RNF115,DDX52,DDX46,UBE2M,TSPAN7,SOS1,DNAJB14,HMGA2,CALB1,ARID3B,ZCCHC14,KPNA4,SCHIP1,ARL4A,DOCK7,SLC5A3,CPEB4,LMO3,GPR137B,IQGAP2,HEPHL1,MTHFD1L,HS6ST1,RHOQ,FBXO33,ZFAND5,ZIC2,TMEM203,SIAE,CBX3,GREM1,FAM204A,ATXN3,SAMD4A,SHISA9,FAM222A,VCPIP1,GPR3,RRAGA,PCYOX1,MEIS2,IL10,GNAQ,DNAJB1,RWDD2B,NCL,TMEM106B,RNF141,PSPC1,SF1,ZNF436,WRNIP1,COL16A1,SCP2,SDF2L1,TOX,HERPUD1,NPTN,CACUL1,DCUN1D1,C3orf14,NPTXR,FBXO47,CDK14,FAM189B |

UEMirs, up-expressed miRNAs in pregnant women with preeclampsia with severe features; UEMirs, down-expressed miRNAs in pregnant women with preeclampsia with severe features.
