# Supplementary figures and images for: Pathogenic mechanisms of preeclampsia with severe features implied by the plasma exosomal mirna profile
Source: Bioengineered. 2021 Dec 9;12(2):9140–9. doi: 10.1080/21655979.2021.1993717 (PMC8810006; doi:10.1080/21655979.2021.1993717)

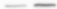

**CD9**

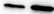

**CD63**

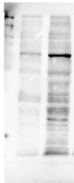

**PLAP**

Supplement: Supplemental Material [file KBIE_A_1993717_SM8811.zip › supplementary figure.pdf]
